# Alterations of Muscle Synergies During Voluntary Arm Reaching Movement in Subacute Stroke Survivors at Different Levels of Impairment

**DOI:** 10.3389/fncom.2018.00069

**Published:** 2018-08-21

**Authors:** Bingyu Pan, Yingfei Sun, Bin Xie, Zhipei Huang, Jiankang Wu, Jiateng Hou, Yijun Liu, Zhen Huang, Zhiqiang Zhang

**Affiliations:** ^1^Sensor Network and Application Research Center, School of Electronic, Electrical and Communication Engineering, University of Chinese Academy of Sciences, Beijing, China; ^2^Rehabilitation Department, Peking University First Hospital, Beijing, China; ^3^School of Electronic and Electrical Engineering, University of Leeds, Leeds, United Kingdom

**Keywords:** Brunnstrom Stage, electromyography, non-negative matrix factorization, muscle synergy, stroke, rehabilitation

## Abstract

Motor system uses muscle synergies as a modular organization to simplify the control of movements. Motor cortical impairments, such as stroke and spinal cord injuries, disrupt the orchestration of the muscle synergies and result in abnormal movements. In this paper, the alterations of muscle synergies in subacute stroke survivors were examined during the voluntary reaching movement. We collected electromyographic (EMG) data from 35 stroke survivors, ranging from Brunnstrom Stage III to VI, and 25 age-matched control subjects. Muscle synergies were extracted from the activity of 7 upper-limb muscles via nonnegative matrix factorization under the criterion of 95% variance accounted for. By comparing the structure of muscle synergies and the similarity of activation coefficients across groups, we can validate the increasing activation of pectoralis major muscle and the decreasing activation of elbow extensor of triceps in stroke groups. Furthermore, the similarity of muscle synergies was significantly correlated with the Brunnstrom Stage (*R* = 0.52, *p* < 0.01). The synergies of stroke survivors at Brunnstrom Stage IV–III gradually diverged from those of control group, but the activation coefficients remained the same after stroke, irrespective of the recovery level.

## Introduction

Movement is one of the basic skills of human beings. We can perform various daily tasks almost effortless. However, the organization of human movement is not straightforward, as it involves complex cooperative interactions between the central nervous system (CNS) and the musculoskeletal system. As each joint of the musculoskeletal system can afford up to 6 degrees of freedom movement, it makes the motor control extremely delicate. Muscle synergy hypothesis, which describes muscle activation of a set of muscles contributing to a particular movement, has been proposed to simplify the motor control (d'Avella et al., 2003). Neural control of movements can be accomplished by a hierarchal framework where muscle synergies are at the bottom and the task-related conceptual parameters are manipulated by higher neural centers (Loeb et al., 1999; Scott, 2004; Todorov et al., 2005). The hypothesis of muscle synergy has been explored by many previous studies. For example, Ting and Macpherson (2005) and Overduin et al. (2008) demonstrated that synergies represented a generalized control strategy in postural control of cats and rhesus macaques. In upper limb movement, the muscle activity can be fully characterized by a relatively limited number of muscle synergies among various motor tasks (d'Avella and Lacquaniti, 2013). Lacquaniti et al. (2012) showed that the muscle activity of human locomotion can be formed by a combination of basic muscle synergies timed at different phases of the gait cycle. These studies showed that muscle synergies could simplify the motor behavior generation and reduce the dimensionality of redundant musculature control problem. Furthermore, muscle synergies are robust and shared across behaviors (d'Avella and Bizzi, 2005; Chia Bejarano et al., 2017; Nazifi et al., 2017; Saito et al., 2018). Dominici et al. (2011) observed that the two basic patterns extracted from newborn babies' locomotor were retained through development and another two new patterns were first revealed in toddlers. The common primitives may relate to a common ancestral neural network.

The purpose of muscle synergies analysis in people who suffered from motor deficits due to inappropriate muscle coordination is to reveal the underlying physiological mechanisms and offer suggestions on efficient recovery process (Safavynia et al., 2011; Casadio et al., 2013). Some studies have been conducted to find out how muscle synergies were affected after stroke. For instance, Clark et al. (2010) illustrated that motor modules in stroke patients locomotion were altered and the number of modules was correlated with biomechanical and clinical walking performance variables. Gizzi et al. (2011) extended the results by analyzing muscle synergies of stroke patients walking at a comfortable speed. They noted that the temporal profile of activation coefficient was preserved while the muscle synergies of the paretic limb were different from those in the contralateral as well as in healthy controls. Similarly, in upper limb motions, Roh et al. (2013, 2015) found alterations in synergy composition from chronic stroke participants. In the study of muscle synergies control during hand-reaching, mildly impaired stroke patients modulated synergies in different ways from the control group (Israely et al., 2018). Another study about the longitudinal changes in upper limb muscle synergies of stroke survivors showed the changes in the number of muscle synergies and the recruitment of muscles during the therapy (Hesam-Shariati et al., 2017). And proper intervention such as physical therapy on the standing-up motion of stroke survivors have been proved to improve the disordered and inadequate muscle synergy structure (Kogami et al., 2018). Furthermore, Cheung et al. (2012) observed that distinct muscle organization patterns such as merging, preservation, and fractionation of muscle synergies occurred after cortical damage. Hashiguchi et al. (2016) also found the merging and fractionation of muscle synergies in subacute stroke patients during gait, and the merging extent was relied on motor function. The abnormal patterns may be explained as compensation strategies of brainstem and spinal control. These results indicated that muscle synergies can provide physiological markers to assess the status of post-stroke survivors. Besides the muscle synergies, data from inertial sensors can also be used as helper methods in rehabilitation process since they can provide precise information on how the limb moves through space (Voinea et al., 2017). However, no former research has examined the potential alterations in structure and recruitment of muscle synergies from stroke patients at different Brunnstrom stages. Brunnstrom Approach is one of the measurements used to assess the motor control restoration throughout the body after stroke, which emphasizes the synergic patterns of movement developed during the recovery (Brunnstrom, 1966, 1970).

In this paper, we examined the alterations of muscle synergy structure and the recruitment patterns in subacute stroke survivors at different Brunnstrom stages during the voluntary reaching movement. Surface electromyography (EMG) and inertial sensor data from 35 stroke survivors ranging from Brunnstrom Stage III to VI and 25 age-matched control subjects were collected. Muscle synergies and recruitment patterns identified by non-negative matrix factorization (NMF) from stroke and healthy groups were compared. This study can provide suggestions on how to make use of the abnormal synergy patterns to accelerate the rehabilitation process by focusing on the exercise of specific muscles.

## Materials and methods

### Participants

Thirty-five patients with stroke and 25 age-matched control subjects were recruited for this study from Peking University First Hospital. Inclusion criteria: (1) diagnosed with stroke for the first time; (2) the duration of stroke was no longer than 6 months; (3) shoulder lift with voluntary at least 30° without help; (4) had no history of other nervous system diseases. The patients enrolled were all suffered cerebral ischemic stroke, and the impairment region was at dominated area of middle cerebral artery. Healthy participants without neurological nor muscular injuries in upper limb were enrolled as control group. The general information for both stroke and control participants are demonstrated in Table 1. Brunnstrom Stages of recruited stroke patients were assessed by professional rehabilitation therapist from Peking University First Hospital. This research has been approved by the Ethics Committee of Peking University First Hospital and all the subjects gave informed consent before experimentations.

**Table 1:** General information of participants.

			**Mean**	***SD***	**Range**
Bruunstrom Stage III (*N* = 15)	Age (year)		59.67	10.57	[38, 83]
	Sex (male/female)	12/3			
	Side affected (left/right)	11/4			
	Fugl-Meyer		15.80	3.92	[9, 23]
Bruunstrom Stage IV (*N* = 7)	Age (year)		57.71	10.61	[40, 72]
	Sex (male/female)	5/2			
	Side affected (left/right)	3/4			
	Fugl-Meyer		30.57	4.69	[25, 38]
Bruunstrom Stage V (*N* = 6)	Age (year)		67.67	10.67	[47, 79]
	Sex (male/female)	5/1			
	Side affected (left/right)	5/1			
	Fugl-Meyer		36.83	5.37	[30, 47]
Bruunstrom Stage VI (*N* = 7)	Age (year)		56.00	9.99	[44, 70]
	Sex (male/female)	5/2			
	Side affected (left/right)	1/6			
	Fugl-Meyer		47.56	3.36	[42, 51]
Control group (*N* = 25)	Age(year)		59.16	9.97	[37, 77]
	Sex (male/female)	13/12			

### Data acquisition

To start with, the subjects sat upright in front of the desk with palms facing the thighs. Then the participants were instructed to perform voluntary upward reaching by flexing shoulder at 90° and thumbs up with each arm and hold on for 2 s. The task was repeated three times for each subject, and with an interval of 3 min. Through the tasks, EMG activity was recorded from 7 upper-limb muscles by ME6000 multi-channel bipolar EMG recording system (Mega Electronics Ltd., Kuopio, Finland) at 1,000 Hz. The recorded muscles were pectoralis major (PECM), upper trapezius (TRA), anterior deltoid (DELA), medial deltoid (DELM), biceps brachii (BIC), triceps brachii (TRI), and brachioradialis (BRAC). Electrodes were placed longitudinally along the muscle fiber directions on corresponding muscles based on the guidelines of the Surface Electromyography for the Non-Invasive Assessment of Muscles (SENIAM) (Hermens et al., 1999). At the same time, the motion information during voluntary reaching task was collected (50Hz) using four MPU-9150 (InvenSense Inc., USA) sensors, each including a tri-axial accelerometer, tri-axial magnetometer and tri-axial gyroscope. The four inertial sensors were attached to the center of the waist as the root, lateral center of upper arm, lateral center of forearm, and lateral center of wrist, respectively.

### Elbow joint angle estimate

In order to observe the change of upper limb behaviors of stroke survivors compared to control subjects, we calculated elbow joint angle from motion data recorded by inertial sensors. We assume that upper arm, forearm, and hand are all rigid bodies, rotating around their corresponding joints. The quaternions were obtained by fusing data from accelerometer, gyroscope and magnetometer according to previous studies (Zhang and Wu, 2011).

(1) $$\begin{array}{r} q_{o}^{BS}={\left(q_{o}^{GB}\right)}^{-1}\cdot q_{o}^{GS} \end{array}$$

(2) $$\begin{array}{r} q_{t}^{GB}=q_{t}^{GS}\cdot q_{t}^{SB}=q_{t}^{GS}\cdot {\left(q_{t}^{BS}\right)}^{-1} \end{array}$$

where the superscript *G* means global, *B* means body, *S* means sensor. The subscript *o* means the initial of time, and *t* means at time t. So $q_{o}^{GB}$ is the quaternion of body in global coordinates in the initial. $q_{o}^{BS}$ remains the same at different time, which means $q_{t}^{BS}=q_{o}^{BS}$. Then the position vector *P* of joint is calculated by:

(3) $$P_{c}^{G}=\;P_{f}^{G}+\;q_{f}^{GB}\;⊗L^{B}⊗\;{\left(q_{f}^{GB}\right)}^{-1}$$

where *c* means child joint and *f* means the father joint. In the hierarchical biomechanical model, shoulder is the father joint of elbow and elbow is the father joint of wrist. *L*^*B*^ is the vector between father joint and child joint. The elbow joint angle was computed as the angle between forearm vectors of start point to endpoint based on hierarchical biomechanical model (Huang et al., 2012).

(4) $$\theta =cos^{-1}\langle \left(P_{wrist}^{e}-\;P_{elbow}^{e}),(P_{wrist}^{s}-\;P_{elbow}^{e}\right)\rangle$$

where superscript of *e* and *s* represent the end and start point. Because in our hierarchical biomechanical model, the elbow joint was moved due to the movement of shoulder joint. Thus, we used the $q_{shoulder}^{-1}$ to eliminate the influence of shoulder joint.

(5) $${q^{\prime }}_{elbow}=\;q_{shoulder}^{-1}\cdot \;q_{elbow}$$

Equation 4 can be simplified by the above equations and we obtained the elbow angle:

(6) $$\theta =cos^{-1}\langle \begin{array}{c} q_{elbow}^{e}⊗L_{fore}^{B}⊗{\left(q_{elbow}^{e}\right)}^{-1},\\ \;q_{shoulder}^{e}⊗L_{fore}^{B}⊗{\left(q_{shoulder}^{e}\right)}^{-1} \end{array}\rangle$$

where $q_{elbow}^{e}$ and $q_{shoulder}^{e}$ are the endpoint of elbow and shoulder quaternion respectively.

### Muscle synergy extraction and analysis

#### Identification of muscle synergies

In order to minimize the disturbances that would affect the EMG signals, preprocessing was conducted before the extraction of muscle synergies. EMG signals were high-pass filtered by window-based finite impulse response filter (50th order, cutoff of 50 Hz), rectified, low-pass filtered by window-based finite impulse response filter (50th order, cutoff of 20 Hz), and integrated over 20-ms intervals sequentially (Cheung et al., 2012). To avoid that the extraction of muscle synergies was biased into describing only the muscles with high-amplitude, we normalized EMG signals of each muscle from each individual within the task by the maximum value, and resampled into 200 points per trial (Burden, 2010).

We modeled EMG patterns (*D*) as linear combinations of few time-invariant muscle synergies (*W*), each recruited by a time-varying coefficient (*c(t)*) (Cheung et al., 2005; Tresch et al., 2006). The recruitment coefficients may reflect the temporal modulation of neural command to muscle synergy and specify how much each synergy contributes to EMG signal of each muscle (Torres-Oviedo et al., 2006). Muscle activation pattern *D* can be expressed as:

(7) $$D(t)=\sum_{i=1}^{N}c_{i}(t)W_{i}$$

where *N* specifies the muscle synergy number. To extract muscle synergies and associated activation coefficients, we performed the algorithm of NMF to the EMG dataset (Lee and Seung, 1999, 2001). In this decomposition process, the elements in synergy and coefficient matrixes were first initialized with random values from a 0 to 1 uniform distribution. Then the values in the two matrixes were iteratively updated using updating rules in Equation (8) until convergence. The synergy extraction process was repeated 50 times for each subject and the synergies with the highest EMG-reconstruction R^2^ was selected for further analyses to maximize the chance of applying R^2^ according with the global optimum of NMF decomposition.

$$\begin{array}{r} W_{ij}\leftarrow W_{ij}\frac{{\left(DC^{T}\right)}_{ij}}{{\left(WCC^{T}\right)}_{ij}} \end{array}$$

(8) $$C_{jk}\leftarrow C_{jk}\frac{{\left(W^{T}D\right)}_{jk}}{{\left(W^{T}WC\right)}_{jk}}$$

#### Estimating muscle synergy number

We used the criterion of variance accounted for (VAF) to determine muscle synergy number, shown as Equation (9) (Cheung et al., 2005; Roh et al., 2011, 2012). VAF was computed from dataset of each subject for 50 times with random initial values of W and C matrix when the number of synergies varied from one to seven. We defined muscle synergy number as the minimum number required to achieve a mean VAF lager than 0.95, which was sufficient to capture the spatial features of the EMG patterns.

(9) $$VAF=1-\frac{\sum_{i=1}^{m}\sum_{j=1}^{t}{\left(EMG_{o}\left(i,j\right)-\;EMG_{r}\left(\;i,j\right)\right)}^{2}}{\sum_{i=1}^{m}\sum_{j=1}^{t}{\left(EMG_{o}\left(\;i,j\right)\right)}^{2}}$$

#### Quantifying similarity of synergies

In order to evaluate the similarity between synergies derived from different dataset, we calculated scalar product between muscle synergies (Tresch et al., 1999). We matched synergies that provided the highest total scalar product to compare individual synergies from two datasets directly, and each synergy was paired only once to the synergy in another dataset. As for the similarity between the activation coefficients, we used another metric of cross-correlation (Hug, 2011; Hug et al., 2011), shown as Equation (10).

(10) $$r_{k}=\frac{\sum_{i=1}^{n-k}\left(X_{i}-\bar{X})(Y_{i+k}-\bar{Y}\right)}{\sqrt{\sum_{i=1}^{n}{\left(X_{i}-\bar{X}\right)}^{2}\sum_{i=1}^{n-k}{\left(Y_{i}-\bar{Y}\right)}^{2}}}$$

where *k* is the time index and $\bar{X},\;\bar{Y}$ are the mean value of *X* and *Y*, separately. The cross-correlation can give information on the possible shift in time and take the temporal profile into account (Dorel et al., 2009). The maximum of the cross-correlation between two activation coefficients where k = 0 was used to assess the differences across signals.

To define a normative synergy template, we first randomly selected one set of three synergies and matched the synergies from remaining synergies then group-averaged to generate mean synergies for each group. The choice of initial dataset has little effect on the group-averaged synergies according to previous studies (Roh et al., 2015). We then used the group mean synergy of control group as the template. For each subject, we calculated the similarities between the three individual synergies and the corresponding template synergy. We excluded the subject's synergy set if the mean similarity was smaller than 0.85, and then re-calculated the means as an updated template, until all the included synergies were similar to the template (Roh et al., 2015). Synergy template for each Brunnstrom stage in stroke survivors was defined by a similar procedure. The normative activation coefficient templates were obtained by group-averaging of the coefficients corresponding to the normative synergy template. Subsequently, we calculated the scalar product as similarity between corresponding synergies in normative synergies of stroke and control to examine the alterations of synergy structure in post-stroke, and the Spearman correlation was calculated to quantify the correlation between similarities and the Brunnstrom Stage. The cross-correlations between activation coefficients were also calculated to test whether alterations also existed in activation coefficients.

#### Merging of synergies

The adapted algorithm proposed by Cheung. (Cheung et al., 2012) was used to identify how the control synergies merged together in the stroke synergy templates. In the model of synergy merging, stroke synergy from each Brunnstrom Stage could be constructed by linear combinations of normative synergy template:

(11) $$w_{i}^{a}\approx \;\sum_{k=1}^{n^{c}}m_{k}^{i}w_{k}^{c},\;m_{k}^{i}\geq 0,i=1,\ldots ,n^{a}$$

where $w_{i}^{a}$ is the *i*th affected-arm synergy from a Brunnstrom stage, $w_{k}^{c}$ is the synergy from the control group's normative synergy template, *n*^*c*^ equals the synergy number of control group, *n*^*a*^ specifies the number of affected-arm synergy from a Brunnstrom stage, and $m_{k}^{i}$ represents the nonnegative coefficient indicating how much the *k*th synergy from normative template contributes to the *i*th synergy's structure in a Brunnstrom stage. A normative synergy was considered as a significant contribution in the merging process if the coefficient $m_{k}^{i}$ was higher than 0.3 (Barroso et al., 2014). Similarity between reconstructed $w_{i}^{a}$ and the initial affected-arm synergy was quantified using scalar product between corresponding columns described as above.

Data analysis were performed using MATLAB 2017a (The Mathworks, Natick, USA). The significant level of statistical tests was fixed at 0.05.

## Results

**Figure 1:**
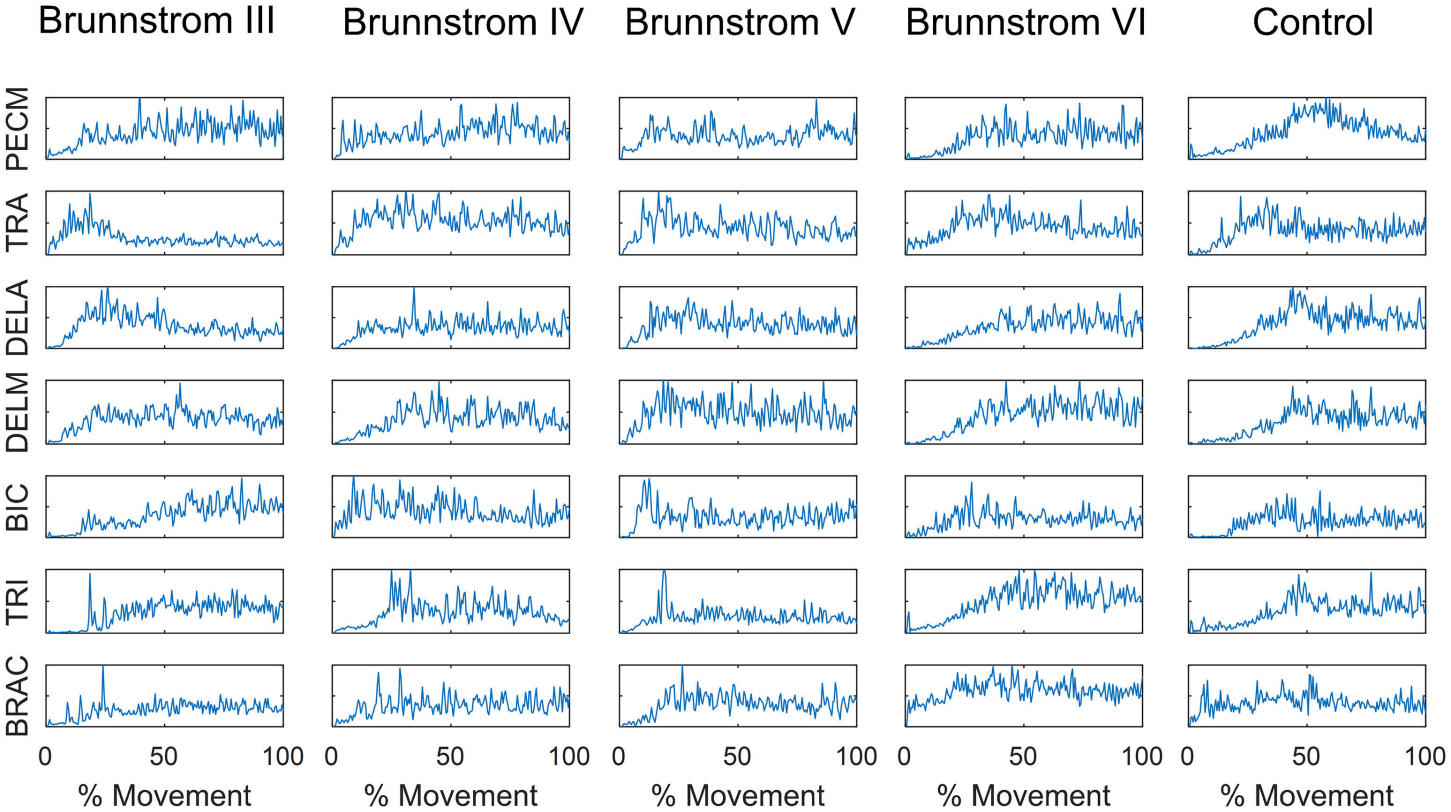
EMG signals of 7 muscles recorded during the reaching task in each Brunnstrom Stage and control group, respectively.

**Figure 2:**
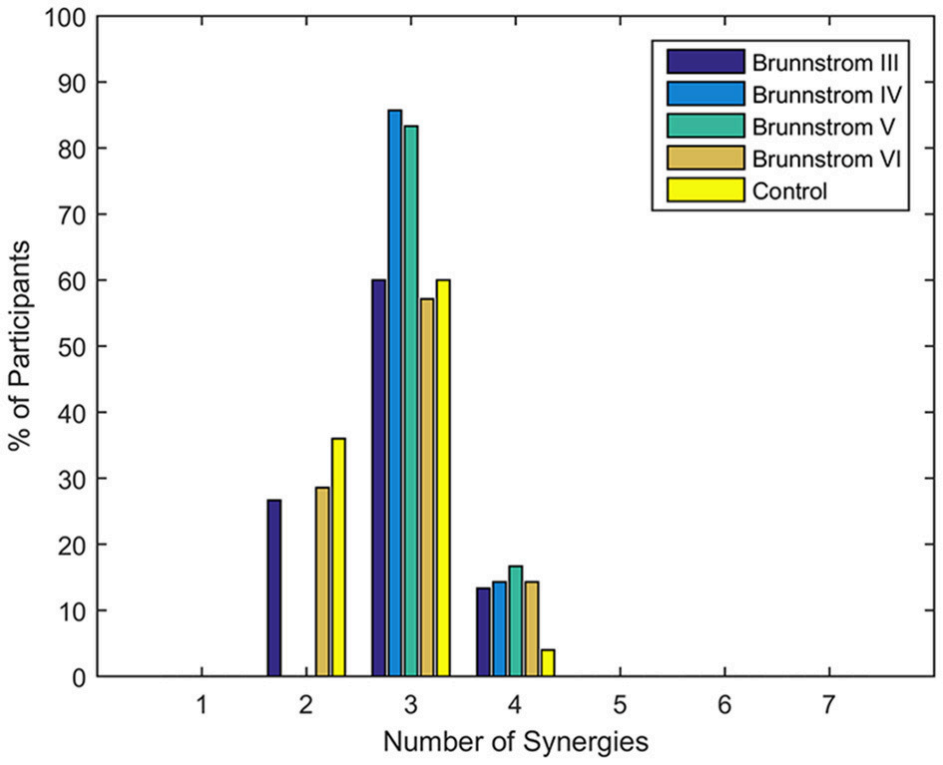
Number of synergies required to reconstruct muscle activation patterns in each Brunnstrom Stage and control group. Typically, 3 synergies were sufficient for all stroke and control groups.

**Figure 3:**
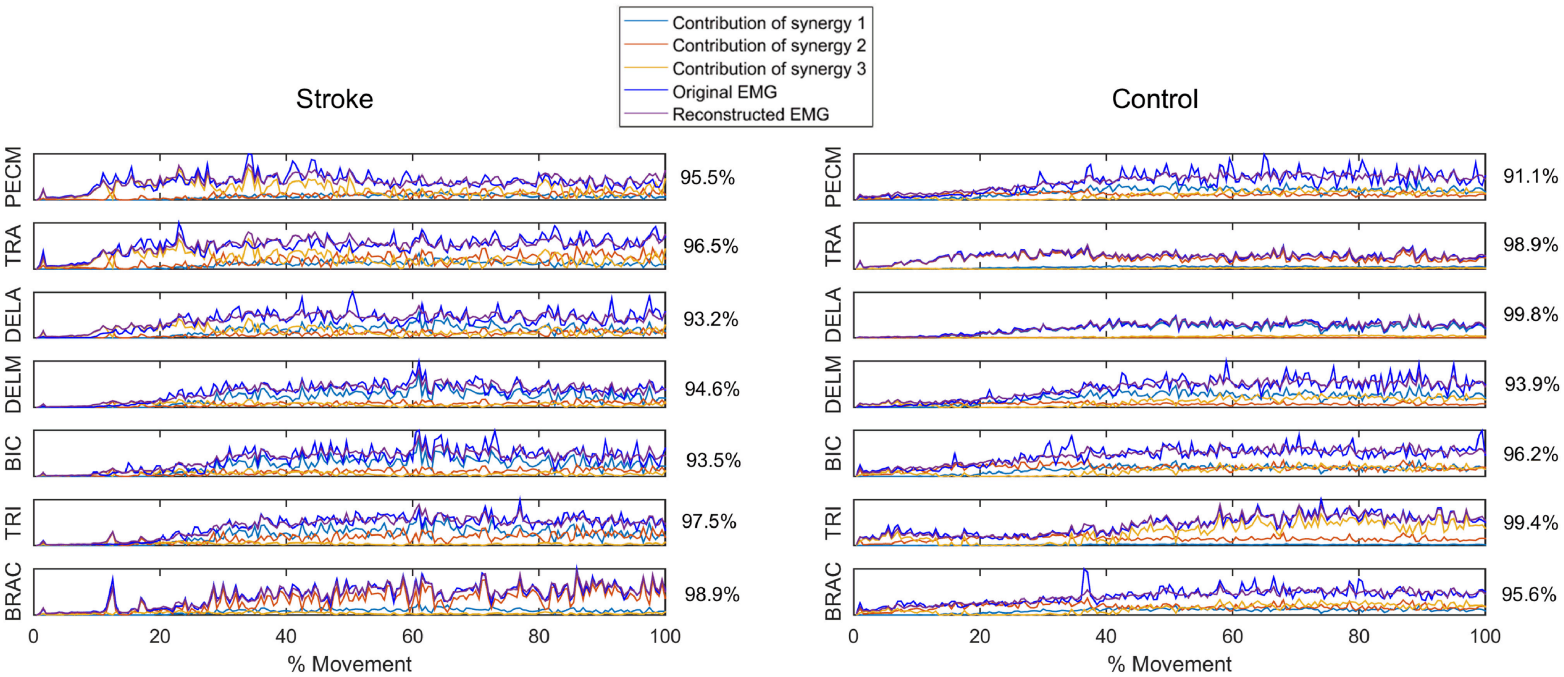
Reconstruction of EMG signal by linear combinations of 3 synergies for a stroke **(Left)** and control **(Right)** subject, respectively. Original EMG, reconstructed data, and contribution of each muscle synergy (lines in different color) to the reconstruction are shown in the figure. Muscle VAF is indicated for each muscle.

**Figure 4:**
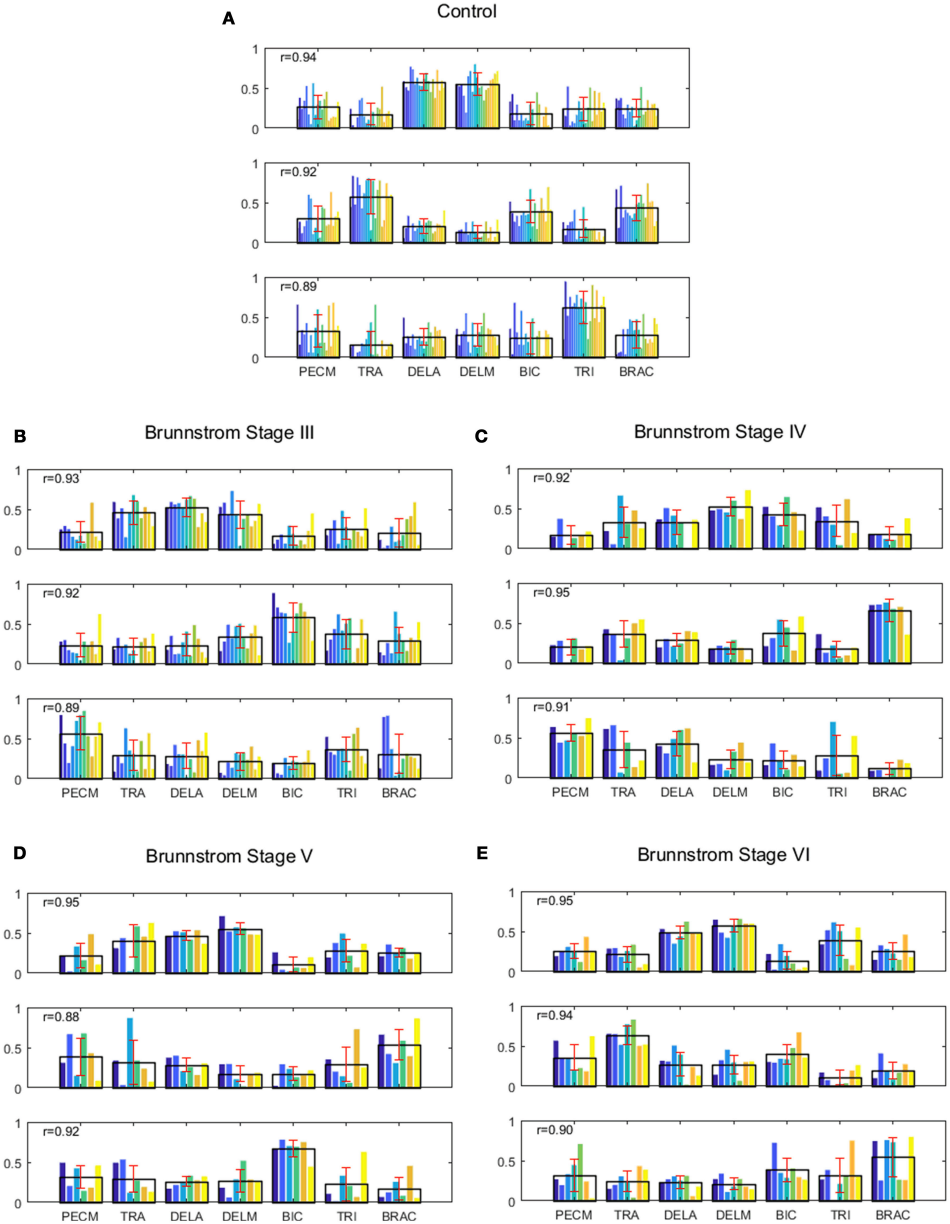
Muscle synergies extracted from control group **(A)** and Brunnstrom Stage III to VI in stroke survivors **(B–E)**. **(A)** In control group, three synergies were identified by the algorithm of NMF from the EMG signals of each subject. Colorized bars show the relative weighting of a muscle and black bars with red represent group means and standard deviations. **(B–E)** In stroke survivors (Brunnstrom Stage III to VI), three synergies were identified from affected-arm of stroke survivors. The r-value in each subpanel represents the mean similarity across all matched pairs of synergies within each group calculated by scalar product.

Figure 1 summarizes the pre-processed EMG activity of seven muscles recorded during the reaching task from a representative subject in each stroke and control group. It can be observed that the difference of EMG signals between control and stroke subjects was mainly in the pattern of trapezius activation. The activation of TRA in stoke survivors at Brunnstrom Stage III–V appeared more highly correlated with DELA and DELM than control group. Muscle synergies were extracted from each EMG dataset to identify underlying intermuscular coordination patterns in subacute stroke survivors. In Figure 2, three synergies were sufficient to reconstruct the original EMG signals in stroke and control groups. More specifically, 2.87 ± 0.64 3.14 ± 0.38, 3.17 ± 0.41, 2.85 ± 0.69, and 2.64 ± 0.57 synergies were identified from four stroke groups and the control group. Figure 3 shows that the combination of muscle synergies and corresponding coefficients can reconstruct the EMG signals excellently, providing high VAF values. Accordingly, three synergies were extracted from each subject for further analysis within and across groups. Figure 4 demonstrates the three muscle synergy patterns from each group of Brunnstrom Stage III to VI and the control, with group mean and standard deviation superposed on individual synergy patterns.

**Figure 5:**
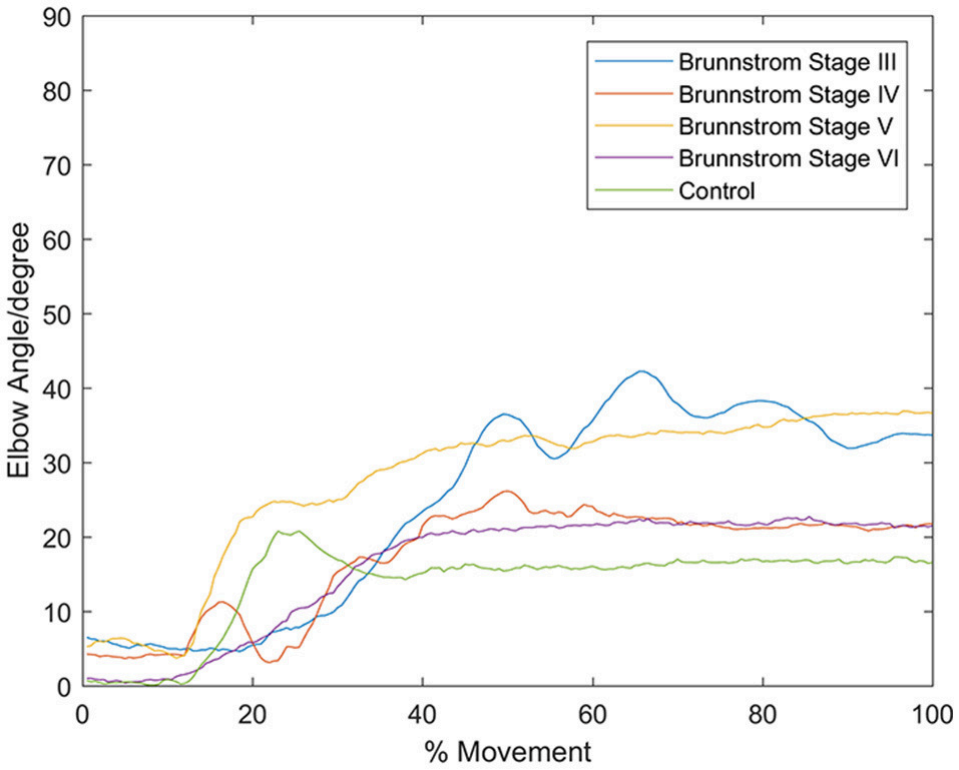
Elbow angle of individual subject from each Brunnstrom Stage and the control group. Stroke patient in Brunnstrom Stage III had the largest elbow joint angle, and had obvious perturbations in the holding phase. The angles in Stage IV to VI were relatively small and smooth compared to Stage III. In control group, there's a peak in the joint angle, which means the subjects bent their elbow and then extended when performed voluntary reaching task.

In all the five groups (Figure 4), the first synergy is dominated by the activation of DELA and DELM, which was referred to as shoulder flexor. Note that in the first synergy from Brunnstrom Stage III, IV, and V, the TRA is also co-activated while in the Brunnstrom VI, the synergy is quite similar to the control. In control group, the second synergy is dominated by activation of TRA, BIC, and BRAC. BIC and BRAC are elbow flexors, and TRA is used for keeping the back straight. The third synergy typically involves activation of TRI, which is the extensor of elbow. When healthy people performed the voluntary upward reaching, they might tend to bend their elbow and extend elbow while raising their hand to get a certain object, which is consistent with the change of elbow joint angle during the task (Figure 5). The other two synergies in Brunnstrom Stage III to VI are consisted of primary activation of shoulder flexor (PECM) or elbow flexors (BIC and BRAC). The increasing activation of PEMC, lack of activation of TRI and abnormality of activation of TRA in post-stroke Brunnstrom Stage III to VI are the most striking differences compared with control group. Since the group mean *r* values in subpanel of Figure 4 are all relatively high, the synergy structure is consistent across subjects within a group. Figure 5 shows the elbow joint angle of subjects in each group. The lack of activation in elbow flexor of triceps in stroke patients (Figure 4) leads to larger elbow angle since they may bend their elbow as a compensation strategy during reaching. Stroke patients in Brunnstrom Stage III have the largest elbow joint angle and obvious perturbations in the holding phase. At Brunnstrom Stage IV and V, spastic muscle movement begins to decline while the voluntary movement becomes more complex, so the angles are relatively small and smooth compared to Stage III. The elbow angles in the control group are the smallest.

**Figure 6:**
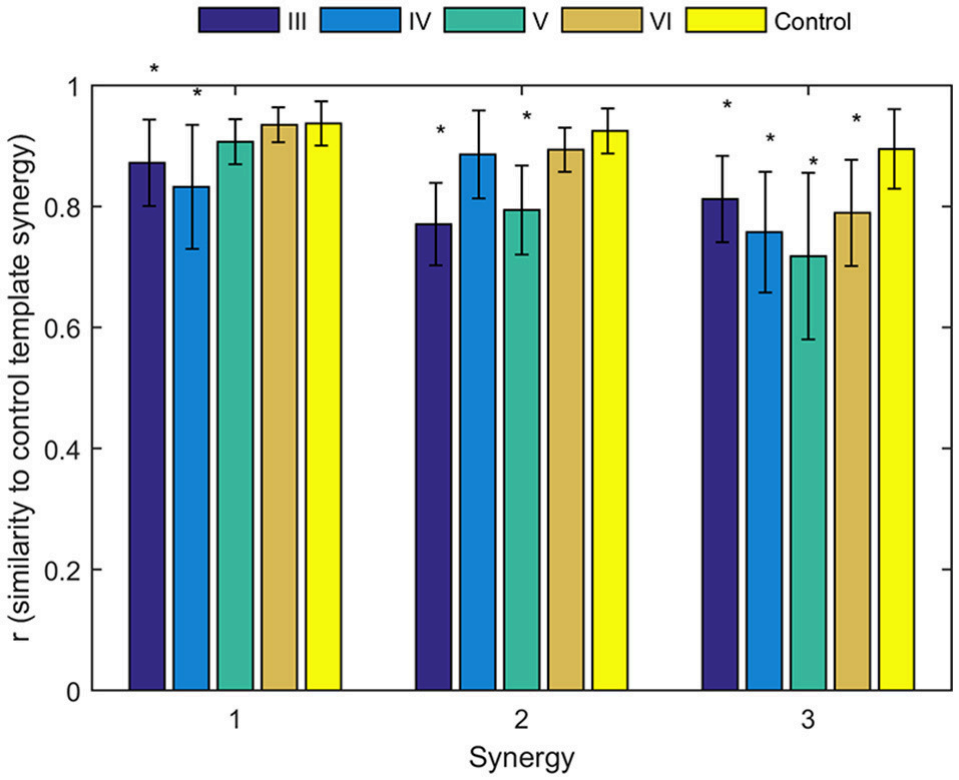
Similarities of synergies compared to control template. The average similarity of the synergies observed for individual subjects in each stroke and control group to the control template (*denotes a significant difference between group means; *p* < 0.05).

To identify alterations of individual synergies in each Brunnstrom Stage, we compute the scalar product between synergies for each subject (including individual control subjects) and corresponding synergy in control template, as summarized by group mean and standard deviation in Figure 6. The asterisks are used to denote those who have significant difference in similarity compared to the control template (two-sample *t*-test, *p* < 0.05). All the three synergies in Brunnstrom Stage III, two of three in Brunnstrom Stage IV and V, one in Brunnstrom VI are significantly different from the control template. This result suggests the alterations in muscle synergy structure of different Brunnstrom Stages. To further quantify the correlation of similarities and the Brunnstrom Stage, we calculate the Spearman correlation between them (*R* = 0.52, *p* = 0.0014) and the similarities correlated significantly with Brunnstrom Stages. Overall, these results reflect the alterations in different Brunnstrom Stage, including the abnormal activation of TRA, the absence of muscle weights of TRI and the increase in PECM across the stroke groups.

**Figure 7:**
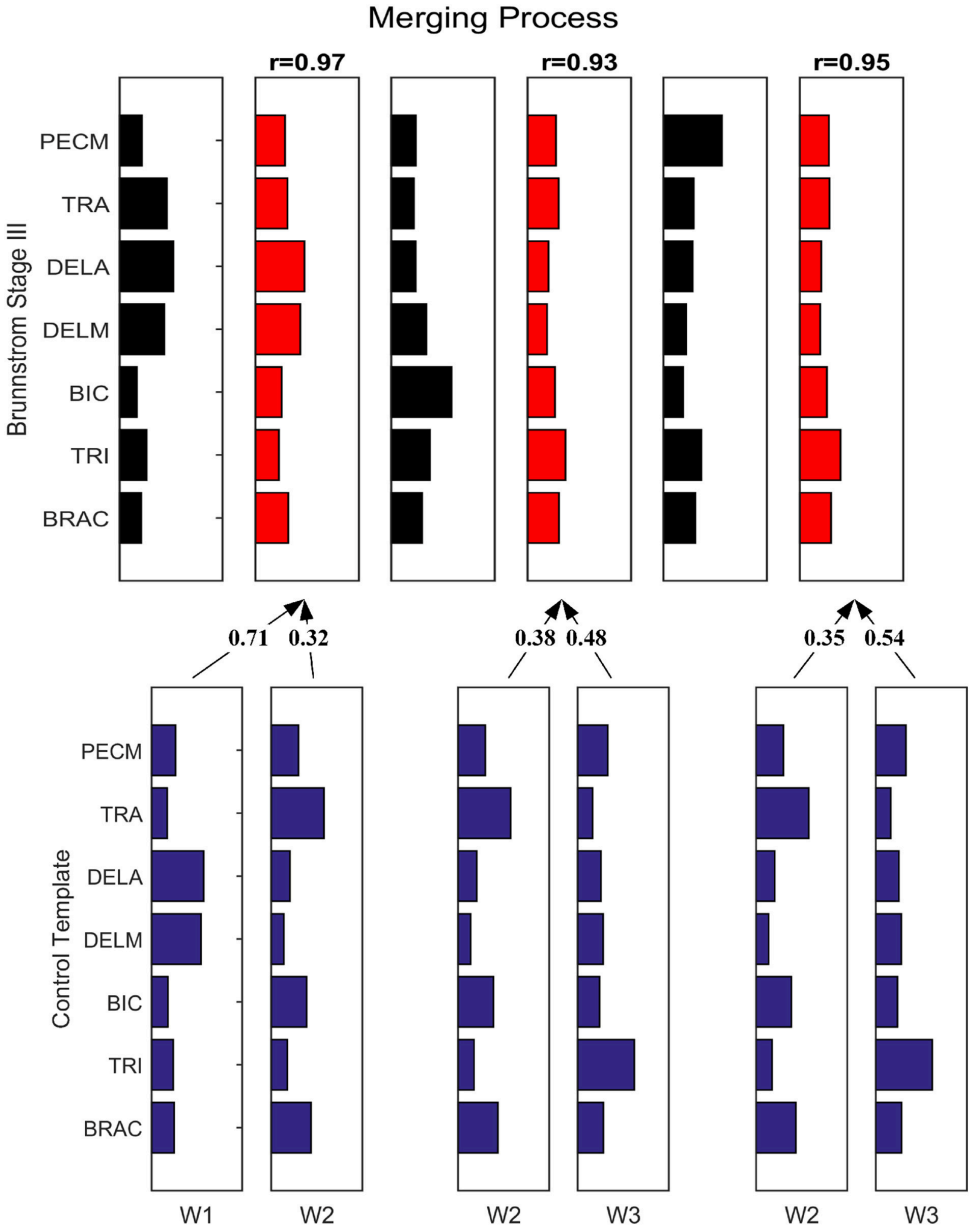
Merging Process. Synergy template of Brunnstrom Stage III explained as a merging of control template. Each of the 3 control synergies was considered to contribute significantly to the merging of a stroke synergy if its merging coefficient was >0.3. Similarity between reconstructed and original cycling synergies was assessed by using the scalar product between corresponding columns.

Figure 7 depicts the process of merging by taking the synergy from Brunnstrom Stage III as an example. The mean synergy of each Brunnstrom Stage can be reconstructed by linear combinations of synergies in control template. The merging coefficients of the merging process are presented in Table 2 calculated according to Equation (11). If the merging coefficient is >0.3, the control synergy is considered to provide significant contribution to the merging of a stroke synergy. Similarities between the original synergies and reconstructed synergies from merging process are summarized in Table 3. The synergies in Brunnstrom Stages can be well reconstructed with all the similarities >0.85. It can be observed from Table 2 and Figure 6 that most of the altered synergies can be reconstructed by the merging process. For example, all the three synergies at Brunnstrom Stage III and the third synergy at Stage IV can be reconstructed by the control template. What's more, the severe group shows a higher degree of synergy merging, which is defined as the mean number of synergies needed in the merging. This trend is in accordance with the outcome in previous research (Cheung et al., 2012). There is also preservation of specific synergies in stroke groups compared with the control template.

**Table 2:** Merging coefficients in each group.

	**Control template**						
	**Synergy 1**	**Synergy 2**	**Synergy 3**		**Synergy 1**	**Synergy 2**	**Synergy 3**
**Brunnstrom Stage III**				**Brunnstrom Stage V**	
Synergy 1	**0.71**	**0.32**	0	Synergy 1	**0.81**	0.23	0
Synergy 2	0.15	**0.38**	**0.48**	Synergy 2	0.12	**0.54**	**0.33**
Synergy 3	0.13	**0.35**	**0.54**	Synergy 3	0.19	**0.57**	0.2
**Brunnstrom Stage IV**				**Brunnstrom Stage VI**	
Synergy 1	**0.52**	0.28	0.23	Synergy 1	**0.79**	0	0.28
Synergy 2	0.18	**0.81**	0.027	Synergy 2	0.18	**0.84**	0
Synergy 3	**0.39**	**0.34**	0.25	Synergy 3	0.049	**0.55**	**0.42**

Bold text indicates the significant contribution in merging process (>0.3)

**Table 3:** Similarity between reconstructed synergies obtained from merging process and corresponding control synergies.

	**Synergy 1**	**Synergy 2**	**Synergy 3**
Brunnstrom Stage III	0.97	0.93	0.95
Brunnstrom Stage IV	0.9	0.93	0.9
Brunnstrom Stage V	0.96	0.95	0.85
Brunnstrom Stage VI	0.98	0.95	0.96

**Figure 8:**
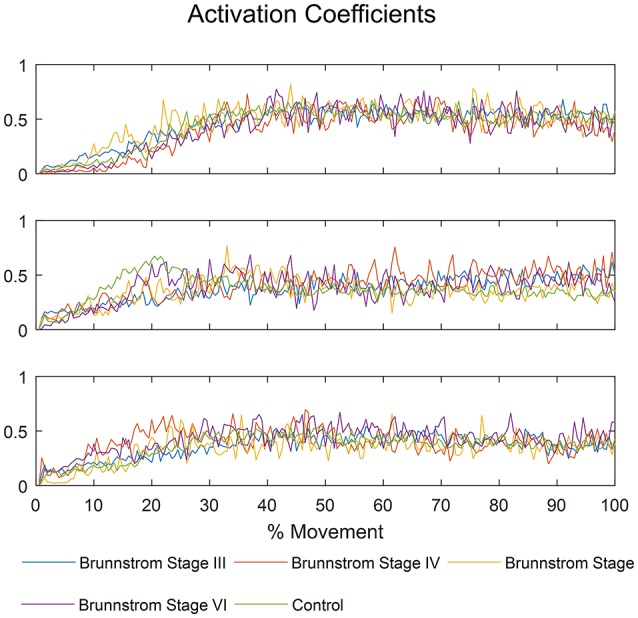
The mean activation coefficients of each Brunnstrom Stage and control group, respectively.

The mean activation coefficients of each group are shown in Figure 8. The similarities of activation coefficients between each Brunnstrom Stage and the corresponding control template are calculated and shown in Table 4. The similarities are all >0.9, which confirms the consistency of the activation coefficients after stroke.

**Table 4:** Cross-correlation of activation coefficients between each Brunnstrom Stage and the corresponding control template.

	**Synergy 1**	**Synergy 2**	**Synergy 3**
Brunnstrom Stage III	0.99	0.92	0.98
Brunnstrom Stage IV	0.98	0.92	0.96
Brunnstrom Stage V	0.98	0.95	0.96
Brunnstrom Stage VI	0.98	0.96	0.98

## Discussion

In this study, we investigated how hemiplegia stroke in different Brunnstrom Stages would affect the structure of muscle synergies and the recruitment patterns during voluntary reaching. For all subjects, three synergies were sufficient to interpret more than 95% of the total variance in EMG signals. We observed the co-activated muscles of trapezius and deltoids and the increased activations of pectoralis major muscle as well as the decreased activation of elbow extensor triceps in stroke groups. The similarity of muscle synergies between stroke and control group was correlated with Brunnstrom Stages. The synergy in post-stroke of each Brunnstrom Stage can be obtained by the merging of control template. What's more, the activation coefficients remained the same after stroke and irrespective of the motor recovery level. Overall, our results indicated that after stroke, different muscle synergies were recruited by similar modulation patterns to complete a movement and the alterations in the structure of muscle synergy in subacute stroke survivors may reflect a compensatory strategy after hemiplegia stroke.

### The number of synergies

NMF was applied to extract muscle synergies from EMG signals, where the identification of synergy numbers was one of the most important steps. However, the optimal number of synergies cannot be calculated automatically throughout all the decomposition methods proposed in the literature. A commonly used method was to choose the lowest number that satisfied the quality of the reconstructed data compared to original recorded EMG under the criterion of VAF/R^2^ (d'Avella et al., 2006; Torres-Oviedo et al., 2006). In this study, the threshold of 95% in VAF was used to identify the number of synergies (Figure 2), according to which three synergies were typically sufficient for most of subjects. In the research of muscle synergy post-stroke during walking, paretic legs needed significantly fewer modules relative to the control (Clark et al., 2010). However, we found stroke patients at Brunnstrom Stage IV and V recruited no less synergies than the control group. This may due to the fact that stroke survivors in this two groups needed more synergies to assist with the reaching movement, such as shrugging their shoulders or bending their elbows. But at Brunnstrom Stage III, the number of synergy was smaller than the stage IV and V, since voluntary movements just started to emerge that they could not coordinate the movement well. Similar results were also observed by Hesam-Shariati et al. (2017) who found that patients with low motor-function needed fewer muscle synergies as the higher number of muscle synergies often reflected greater movement complexity. And at Stage VI, the synergy number was similar to the control, while movement ability was also similar to the control group.

### Implications for neurorehabilitation

Alterations of synergies in subacute stroke patients were observed and the similarities compared to the control group were correlated with Brunnstrom Stages. Similar results were observed in the studies of human locomotion and isometric hand tasks where alterations in muscle synergies were most prominent in severely impaired stroke survivors, and lesser in mild-to-moderate impaired subjects (Clark et al., 2010; Lee et al., 2013). Thus, muscle synergy analysis is a useful method to identify abnormalities in muscle coordination. The altered structure of muscle synergy could reflect changes in neural excitability and affect the muscle coordination patterns (Dietz and Sinkjaer, 2007). The study of muscle synergies may provide a basis for the development of training protocols addressing impaired motor coordination (Safavynia and Ting, 2012). Individualized therapeutic strategies can be developed by focusing on abnormal synergy patterns to accelerate the rehabilitation process. In addition, assistive approaches, such as robot-assisted technology and functional electrical stimulation can be beneficial to the restoration of muscle synergy structure and recruitment. Tracking of the development of abnormal muscle synergies during recovery may also provide a new perspective on stroke rehabilitation.

### Limitations and future work

This study focuses on the alterations of muscle synergies at different Brunnstrom Stages, but there are limitations that need to be improved in further research. First, we would include more stroke patients in different impairment levels to provide a more convincing result and deeper understanding about the neurophysiological explanation of muscle synergies. Besides, the stroke duration of participants was no longer than 6 months, but the time since stroke onset was not considered in this study. Researches have proved that cortical reorganization occurred in stroke patients receiving a rehabilitation therapy (Sawaki et al., 2014; Shimamura et al., 2017) and the degree of reorganization was related to the duration of post-stroke. Overt and covert exercise of stroke patients can activate the sensorimotor cortices, which may influence the recruitment of muscle synergies (Szameitat et al., 2012). In the future, we should consider the role of timing on the alterations in muscle synergies and the merging process since cortical reorganization, which are common after stroke onset, can influence temporal processing.

## Author contributions

BP, YS, BX, ZhiH, JW, JH, YL, ZheH, and ZZ conceived and designed the experiments. BP and YL performed the experiments. BP analyzed the data and wrote the paper. YS, BX, ZhiH, JW, JH, YL, ZheH, and ZZ revised the paper.

### Conflict of interest statement

The authors declare that the research was conducted in the absence of any commercial or financial relationships that could be construed as a potential conflict of interest.
